# Dose-Dependent Effects of Selenium Methionine Supplementation on Functional, Structural, and Physiological Characteristics of Rooster Semen During Liquid Storage at 25 °C

**DOI:** 10.3390/vetsci13040334

**Published:** 2026-03-30

**Authors:** Areej Arif, Nousheen Zahoor, Aqsa Sadiq, Tariq Sohail, Meihui Tang, Liyue Dong, Jianqiang Tang, Sardar Zarq Khan, Guojun Dai

**Affiliations:** 1 College of Animal Science and Technology, Yangzhou University, Yangzhou 225000, China; areej.arif.2024@gmail.com (A.A.); nousheenzahoor17@gmail.com (N.Z.); drtariqsohail34@yahoo.com (T.S.); m19856015485@163.com (M.T.); 13365115757@163.com (L.D.); tangjian62538@163.com (J.T.); 2 College of Veterinary Medicine, Yangzhou University, Yangzhou 225000, China; aksaasadiq@gmail.com; 3 Riphah College of Veterinary Science, Riphah University, Lahore 05450, Pakistan; zarqniazi22@gmail.com

**Keywords:** selenium methionine, rooster semen, liquid storage, oxidative stress, antioxidant supplementation, computer-assisted sperm analysis (CASA), dose–response modeling, sperm membrane integrity

## Abstract

The preservation of rooster semen quality during short-term liquid storage remains a challenge in poultry reproductive biotechnology because sperm cells rapidly lose functional competence under ambient conditions. This deterioration is largely associated with oxidative stress and lipid peroxidation of sperm membranes, which are particularly vulnerable in avian species due to their high polyunsaturated fatty acid content and limited cytoplasmic antioxidant defenses. Selenium is an essential trace element involved in cellular antioxidant protection through its incorporation into several selenoproteins that regulate redox balance and protect cellular structures from oxidative injury. The present study evaluated the effects of selenium methionine supplementation on rooster semen quality during liquid storage at 25 °C. Semen was diluted using a standard poultry semen extender composed of sodium glutamate, glucose, potassium acetate, magnesium acetate, and potassium citrate. Selenium methionine was incorporated into the semen extender at concentrations of 0.5%, 1%, and 2% (*w*/*v*) at the time of semen dilution prior to storage. Semen quality was assessed at 0, 4, 8, 12, and 24 h of storage. Functional parameters, including total sperm motility, sperm viability, and dead sperm percentage, together with kinematic variables (VSL, VCL, VAP, ALH, LIN, and STR), were analyzed using computer-assisted sperm analysis (CASA). Structural integrity was evaluated through acrosome and plasma membrane integrity tests, while sperm physiological status and apoptotic progression were assessed using Annexin V-FITC/propidium iodide flow cytometry. Significant effects of storage time, selenium methionine concentration, and their interaction were observed for multiple semen quality parameters (*p* < 0.05). Among the tested concentrations, supplementation with 0.5% selenium methionine consistently produced the most favorable results, maintaining higher sperm motility, viability, and membrane integrity while reducing dead sperm percentage and apoptotic progression during storage, with protective effects particularly evident at 8, 12, and 24 h compared with the control and higher concentrations. Polynomial contrast analysis indicated predominantly non-linear dose–response relationships, with quadratic and cubic components providing the best model fit (R^2^ = 0.90–0.99; *p* < 0.0001), suggesting a hormetic antioxidant effect. Overall, these findings indicate that selenium methionine supplementation in semen extender improves the stability of rooster semen during short-term liquid storage at ambient temperature, with 0.5% showing the most consistent protective effects among the concentrations evaluated.

## Simple Summary

Maintaining semen quality during short-term storage remains a major challenge in poultry production, particularly in artificial insemination systems. Sperm cells are highly susceptible to oxidative damage at ambient temperature, which reduces motility, viability, and fertilizing capacity. This study investigated the protective effect of selenium methionine, a natural antioxidant, on rooster semen during storage at 25 °C. Semen samples were supplemented with different concentrations of selenium methionine and evaluated over 24 h for motility, viability, structural integrity, and cellular status. Semen quality declined over time in all groups; however, selenium methionine supplementation significantly reduced this deterioration. Among the tested concentrations, 0.5% consistently provided the greatest protective effect by maintaining higher sperm motility and viability, preserving membrane and acrosome integrity, and reducing apoptotic and necrotic changes. Higher concentrations showed comparatively lower effectiveness, indicating a non-linear dose–response relationship rather than a simple concentration-dependent effect. These findings highlight the importance of optimal antioxidant dosing for semen preservation. Overall, selenium methionine supplementation improved semen stability during short-term storage at room temperature. This approach has practical relevance for artificial insemination programs, particularly in resource-limited settings where refrigeration is not readily available, and may contribute to improved reproductive efficiency in poultry production.

## 1. Introduction

Artificial insemination (AI) is routinely employed in commercial poultry production to enhance reproductive efficiency, facilitate genetic selection, and improve biosecurity management [1,2]. However, the effectiveness of AI programs is significantly limited by the ability to maintain semen quality during short-term liquid storage. Rooster spermatozoa are particularly vulnerable to environmental stressors, particularly oxidative disturbances and temperature-mediated metabolic alterations, which lead to progressive losses in motility, membrane integrity, and fertilizing capacity [3,4].

Oxidative stress is widely recognized as a principal mechanism underlying sperm deterioration during storage. Avian spermatozoa possess membranes containing high proportions of long-chain polyunsaturated fatty acids (PUFAs), which render them susceptible to lipid peroxidation [5,6]. During liquid storage at ambient temperature (25 °C), mitochondrial respiration continues, generating reactive oxygen species (ROS) through electron leakage from the electron transport chain [7,8]. Excessive ROS accumulation damages phospholipids, collapses mitochondrial membrane potential, impairs ATP production, alters flagellar movement, and activates apoptotic pathways [9,10]. While sperm cell possesses endogenous antioxidant systems such as superoxide dismutase, catalase, and glutathione peroxidases (GPxs), their limited cytoplasmic volume restricts antioxidant defense capacity [5,11,12].

Selenium is an essential trace element in male reproductive function. It is incorporated into selenoproteins as selenocysteine, particularly glutathione peroxidases (GPx1 and GPx4), which neutralize hydrogen peroxide and lipid hydroperoxides, thereby limiting oxidative damage [11,13]. GPx4 plays both structural and functional roles in spermatozoa, contributing to the formation of the mitochondrial sheath and supporting sperm maturation [13,14]. Glutathione peroxidases also play a central role in maintaining cellular redox balance by reducing hydrogen peroxide and lipid hydroperoxides, thereby protecting cellular lipids, proteins, and DNA from oxidative damage and supporting redox-sensitive cellular signaling. Insufficient selenium availability has been associated with impaired spermatogenesis, reduced motility, and increased susceptibility to oxidative stress. Selenium is incorporated into several selenoproteins involved in antioxidant defense and redox regulation, including glutathione peroxidases, thioredoxin reductases, selenoprotein P, and methionine-R-sulfoxide reductase, which collectively contribute to cellular protection against oxidative stress [15,16].

Among selenium supplements, selenium methionine represents an organic form characterized by high bioavailability and metabolic stability [17]. Its structural similarity to methionine allows it to be non-specifically incorporated into general protein pools in place of methionine, serving as a functional antioxidant reserve [18]. However, selenium possesses a narrow therapeutic window; excessive intake may shift redox balance toward pro-oxidant activity [15,19]. Therefore, precise determination of the optimal concentration is essential.

Although dietary selenium supplementation in poultry nutrition has been widely investigated [5,20], evidence exists regarding the direct supplementation of selenium methionine into semen extenders. Furthermore, systematic dose-dependent evaluation under ambient storage conditions using integrated functional (CASA), structural (acrosome and plasma membrane), and physiological evaluation of apoptotic progression assessments combined with polynomial dose–response modeling remains scarce.

Therefore, the present study aimed to evaluate the effects of graded concentrations of selenium methionine on rooster semen quality during liquid storage at 25 °C. The study investigated functional, structural, and physiological indicators of sperm integrity and explored the dose–response relationship of selenium methionine supplementation during storage.

## 2. Materials and Methods

### 2.1. Ethical Statement

All animal experiments and procedures were conducted in accordance with the guidelines for the care and use of animals in research. The birds were handled with minimal stress and discomfort during semen collection and handling procedures. The experimental protocol was approved by the Institutional Animal Care and Use Committee (IACUC) of Yangzhou University, Yangzhou, China. The animal experiment protocols were conducted under the institutional animal use license issued by the Animal Welfare Committee of Yangzhou University (permit number: SYXK (Su) IACUC 2012-0029).

### 2.2. Experimental Design

The research was designed to investigate the effect of selenium methionine addition to semen diluent on the quality of rooster semen stored under liquid and varying temperatures. Four groups of roosters were prepared for the experiments:

- Control group (extender without selenium methionine);
- Selenium methionine 0.5% (*w*/*v*);
- Selenium methionine 1% (*w*/*v*);
- Selenium methionine 2% (*w*/*v*).

Semen was diluted and stored at 25 °C (ambient temperature) for 24 h. The experiments were conducted at 0, 4, 8, 12, and 24 h. This was performed according to the protocols for short-term storage of semen for roosters [21,22].

The concentrations of selenium methionine used in the experiments are based on previous research on the redox-modulating and hormetic effects of selenium compounds [23,24]. Three experiments (*n* = 3) with pooled semen samples were conducted.

### 2.3. Experimental Birds and Semen Collection

A total of 15 healthy adult roosters of comparable age and physiological status were used for semen collection in this study. The birds were maintained under uniform management conditions as recommended for poultry reproductive management [25,26]. All roosters were obtained from the same breeding facility and belonged to the commercial crossbred roosters. The birds were provided with unlimited access to food and water throughout the experiment.

Semen was collected using the abdominal massage technique, which is a well-established and widely accepted method in poultry semen research [21,22]. Semen samples with abnormal coloration, contamination, or poor motility were rejected to ensure standardization of quality [25].

To minimize individual variations and enhance experiment reproducibility, a pooled semen sample from different males was employed prior to treatment assignment, as suggested for avian semen preservation research [27,28].

### 2.4. Preparation of Selenium Methionine Treatments

Analytical-grade selenium methionine (SM) was accurately weighed and dissolved in the semen extender to prepare treatment solutions of 0.5%, 1%, and 2% (*w*/*v*), corresponding to 0.5 g, 1 g, and 2 g of SM per 100 mL of extender, respectively. The solutions were prepared under sterile laboratory conditions to prevent contamination. To ensure complete dissolution and homogeneity, selenium methionine was gradually added to the extender and mixed thoroughly using gentle agitation until a clear and uniform solution was obtained. Fresh selenium methionine solutions were prepared separately for each biological replicate to avoid potential oxidation and degradation, thereby maintaining chemical stability and consistency across experiments. Selenium methionine, as an organic selenium source, is known for its high bioavailability and metabolic stability, as it can be non-specifically incorporated into protein pools, contributing to sustained antioxidant activity [29,30]. Therefore, careful preparation of fresh solutions was essential to preserve functional integrity throughout the experimental procedure [31].

### 2.5. Semen Dilution and Storage

Fresh semen samples were diluted at a ratio of 1:10 (semen:extender, *v*/*v*) using a standard poultry semen extender composed of sodium glutamate, glucose, potassium acetate, magnesium acetate, and potassium citrate. The extender was freshly prepared prior to use under sterile laboratory conditions to ensure consistency and minimize contamination [22,27].

Selenium methionine was incorporated into the extender at the time of semen dilution to achieve the desired final concentrations. No additional supplementation was applied during subsequent storage or analysis time points, ensuring that all observed effects were attributable to the initial treatment.

Following dilution, semen samples were gently mixed to ensure uniform distribution of sperm cells and treatment solutions. The diluted samples were then aliquoted into sterile 1.5 mL polypropylene microcentrifuge tubes and maintained at 25 °C for up to 24 h.

The selected storage temperature (25 °C) was chosen to simulate practical handling and transport conditions commonly encountered in poultry artificial insemination systems, where refrigeration may not always be available. This condition also allows the assessment of oxidative stress–induced deterioration under moderate metabolic activity [32,33,34]. Semen quality was evaluated at 0, 4, 8, 12, and 24 h of storage. The evaluated parameters included functional parameters (total sperm motility, sperm viability, and dead sperm percentage), kinematic parameters (VSL, VCL, VAP, ALH, LIN, and STR), and structural integrity parameters (acrosome integrity and plasma membrane integrity).

### 2.6. Computer-Assisted Sperm Analysis (CASA)

Sperm motility and kinematic parameters were analyzed using a computer-assisted sperm analysis (CASA) system (Mailiang ML-608JZ V5, Nanning Mailiang Technology Co., Ltd., Nanning, China). CASA was performed according to internationally accepted guidelines for sperm motility evaluation [35,36,37,38,39]. The following parameters were recorded:

- Sperm motility (%);
- Mortality (%);
- Sperm viability (%);
- Straight-line velocity (VSL; μm/s);
- Curvilinear velocity (VCL; μm/s);
- Average path velocity (VAP; μm/s);
- Amplitude of lateral head displacement (ALH; μm);
- Linearity (LIN);
- Straightness (STR).

Dead sperm percentage was determined as the proportion of non-motile spermatozoa detected by the CASA system and was used as an indicator of sperm mortality during storage. Sperm viability represented the percentage of live sperm cells relative to the total sperm population. Velocity parameters (VSL, VCL, and VAP) were expressed in μm/s, ALH in μm, while LIN and STR were expressed as dimensionless ratios calculated from CASA-derived velocity measurements. The CASA system used in this study was configured to provide total motility and the selected kinematic variables evaluated herein; progressive motility and subpopulation categories such as fast- and slow-moving spermatozoa were not included in the output settings used for analysis.

For each sample, at least five microscopic fields were analyzed using a Makler counting chamber (10 μm depth), and approximately 5 μL of diluted semen was loaded for each measurement [40].

### 2.7. Assessment of Acrosome Integrity

Acrosome integrity was evaluated using Giemsa staining under light microscopy as previously described [41,42]. At least 200 spermatozoa were examined per slide to calculate the percentage of sperm with intact acrosomes [8,9].

### 2.8. Assessment of Plasma Membrane Integrity

Plasma membrane integrity was evaluated using the hypo-osmotic swelling test (HOST) as previously described [43]. The percentage of spermatozoa with intact plasma membranes was calculated relative to the total number of sperm cells examined [44,45,46].

### 2.9. Flow Cytometry (Annexin V-FITC/PI Staining)

Flow cytometric analysis was performed using a BD FACSCalibur flow cytometer (Becton Dickinson, San Jose, CA, USA). The physiological state of sperm was analyzed for apoptosis by Annexin V-FITC/Propidium Iodide staining, following standard protocols for apoptosis detection [47,48].

The different states of cells are distinguished by this assay based on the following criteria:

- Viable cells;
- Early apoptotic cells;
- Late apoptotic cells;
- Necrotic cells.

Annexin V specifically stains phosphatidylserine, which is externalized during early apoptosis, and PI stains cells with compromised membranes [49,50].

### 2.10. Statistical Analysis

All data were analyzed with the help of SPSS software 13.0 for windows (SPSS Inc., Chicago, IL, USA) and GraphPad Prism version 9.0 (GraphPad Software, San Diego, CA, USA). Data are presented as mean ± SEM. Two-way ANOVA with the fixed effects of treatment and storage time was used to analyze the data. Interaction between the two factors (treatment × time) was also tested. If any significant effect or interaction effect was observed, Tukey’s post hoc multiple comparison tests were used to assess the data at each time interval. The significance level for the tests was set at *p* < 0.05. Normality of the data was tested with the Shapiro–Wilk normality test [51,52], and the homogeneity of variances was evaluated using Levene’s test [52]. All datasets met parametric assumptions (*p* > 0.05). Polynomial contrast analysis (linear, quadratic, and cubic components) was conducted to evaluate dose–response relationships between selenium methionine concentration (0%, 0.5%, 1%, and 2%) and semen quality parameters. Model fit was assessed using the coefficient of determination (R^2^) [53,54]. Pearson correlation analysis was conducted to explore the relationships between functional (total sperm motility, dead sperm percentage, and sperm viability), kinematic (VSL, VCL, VAP, ALH, LIN, and STR), and structural (acrosome and plasma membrane integrity) parameters [55]. Correlation coefficients were calculated from treatment by time mean values (*n* = 20 observations). Significance was set at *p* < 0.05. All statistical analyses were performed in accordance with standard guidelines for biological experiments. The choice of statistical tests and data presentation methods was based on the nature of the dataset, with multiple group comparisons analyzed using ANOVA with post hoc testing and pairwise comparisons evaluated separately. Data presentation (mean ± SEM or mean ± SD) and significance notation (letters or asterisks) were selected accordingly to ensure accurate interpretation and clarity.

## 3. Results

### 3.1. Effects of Selenium Methionine on CASA Parameters at 25 °C

**Figure 1:**
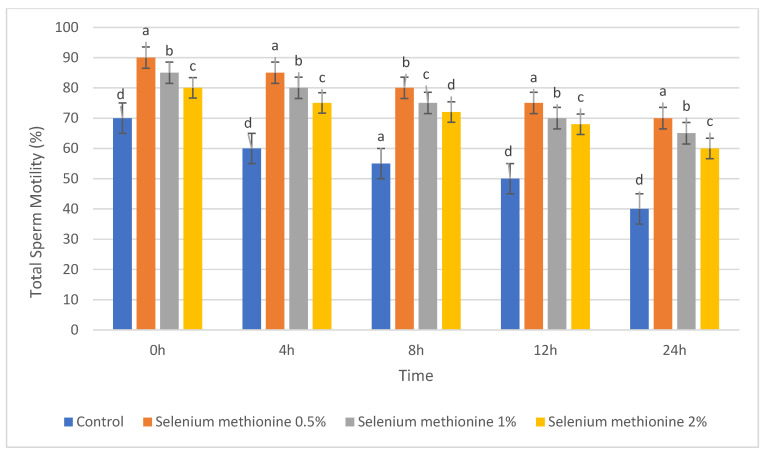
Changes in total sperm motility (%) of rooster semen during storage at 25 °C following selenium methionine supplementation. Data are presented as mean ± SEM (*n* = 3 experimental replicates). Different superscript letters indicate significant differences among treatments at the same time point (*p* < 0.05).

**Figure 2:**
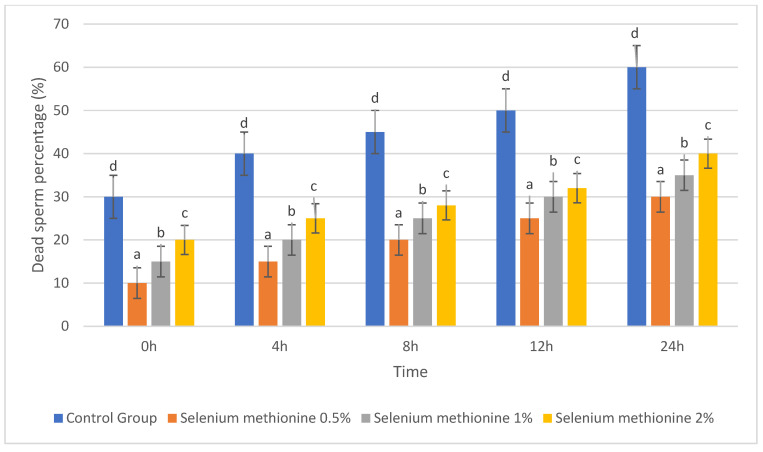
Changes in dead sperm percentage (%) of rooster semen during storage at 25 °C following selenium methionine supplementation. Values are presented as mean ± SEM (*n* = 3 experimental replicates). Different superscript letters indicate significant differences among treatments at the same time point (*p* < 0.05).

**Figure 3:**
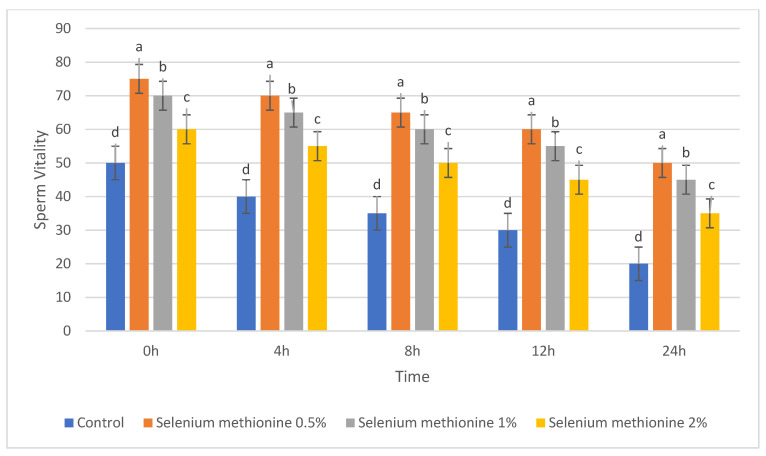
Changes in sperm viability (%) during ambient storage (25 °C) after selenium methionine supplementation. Values are presented as mean ± SEM (*n* = 3 experimental replicates). Different superscript letters indicate statistically significant differences among treatments at the same time point (*p* < 0.05).

**Figure 4:**
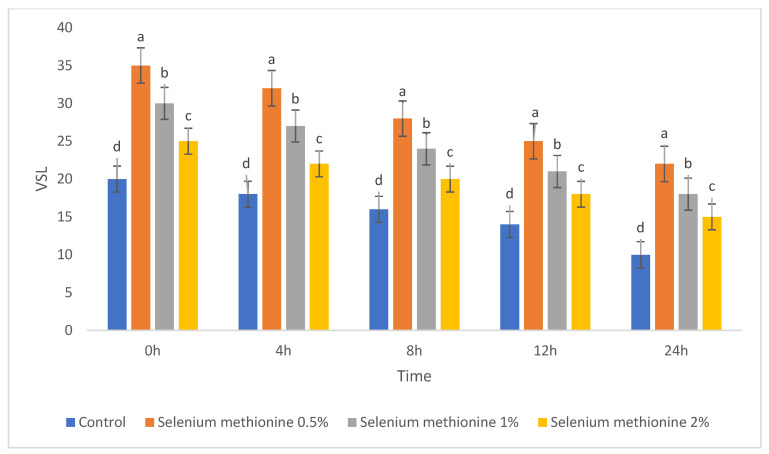
Changes in straight-line velocity (VSL, µm/s) of rooster sperm during storage at 25 °C following selenium methionine supplementation. Data are presented as mean ± SEM (*n* = 3 experimental replicates). Different superscript letters indicate significant differences among treatments at the same time point (*p* < 0.05).

**Figure 5:**
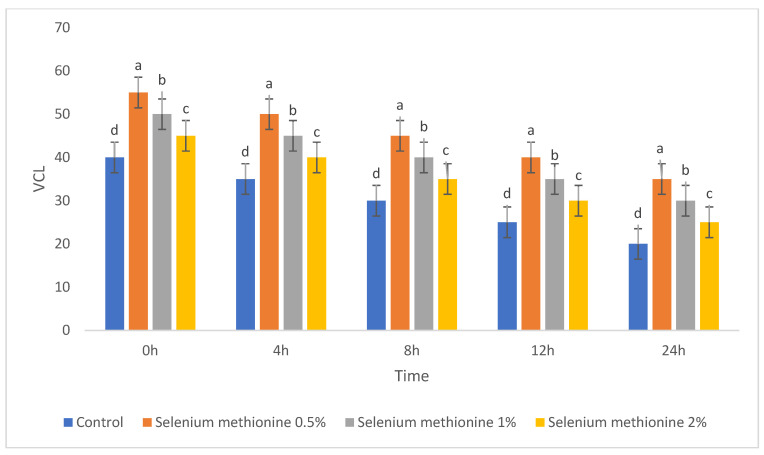
Changes in curvilinear velocity (VCL, µm/s) of rooster sperm during storage at 25 °C following selenium methionine supplementation. Data are presented as mean ± SEM (*n* = 3 experimental replicates). Different superscript letters indicate significant differences among treatments at the same time point (*p* < 0.05).

**Figure 6:**
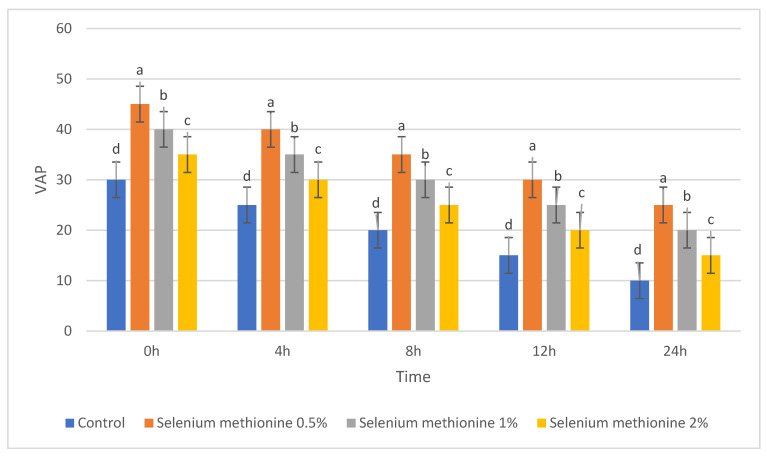
Changes in average path velocity (VAP, µm/s) of rooster sperm during storage at 25 °C following selenium methionine supplementation. Data are expressed as mean ± SEM (*n* = 3 experimental replicates). Different superscripts indicate significant differences among treatments at the same storage time (*p* < 0.05).

**Figure 7:**
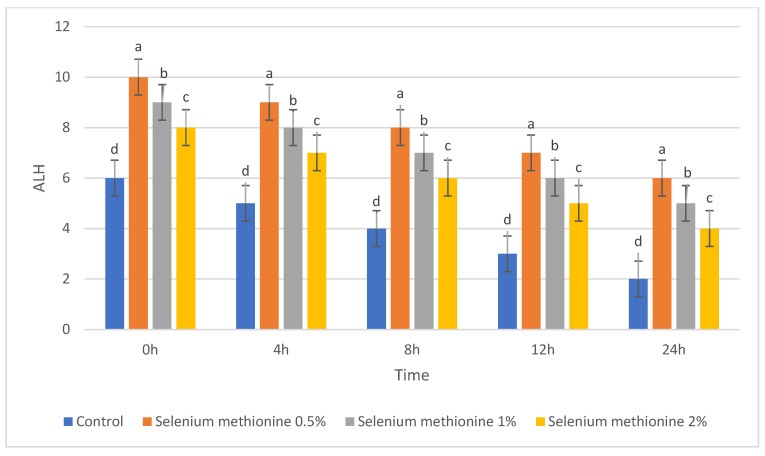
Changes in amplitude of lateral head displacement (ALH, µm) of rooster sperm during storage at 25 °C following selenium methionine supplementation. Data are presented as mean ± SEM (*n* = 3 experimental replicates). Different superscript letters indicate significant differences among treatments at the same time point (*p* < 0.05).

**Figure 8:**
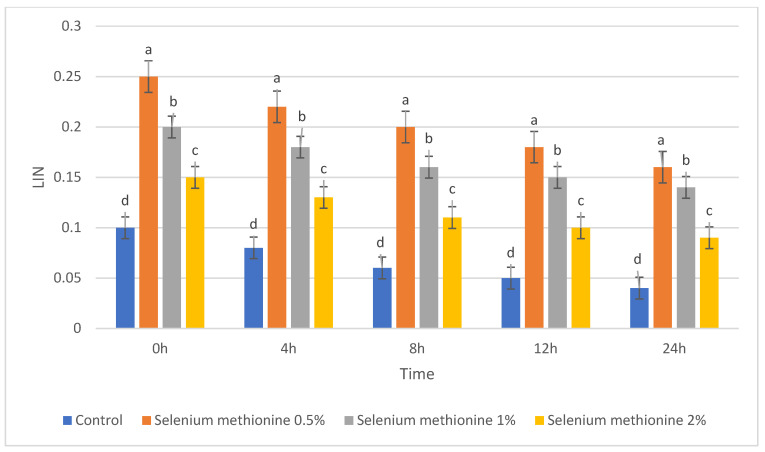
Changes in linearity index (LIN) of rooster sperm during storage at 25 °C following selenium methionine supplementation. Data are presented as mean ± SEM (*n* = 3 experimental replicates). Different superscript letters indicate significant differences among treatments at the same time point (*p* < 0.05).

**Figure 9:**
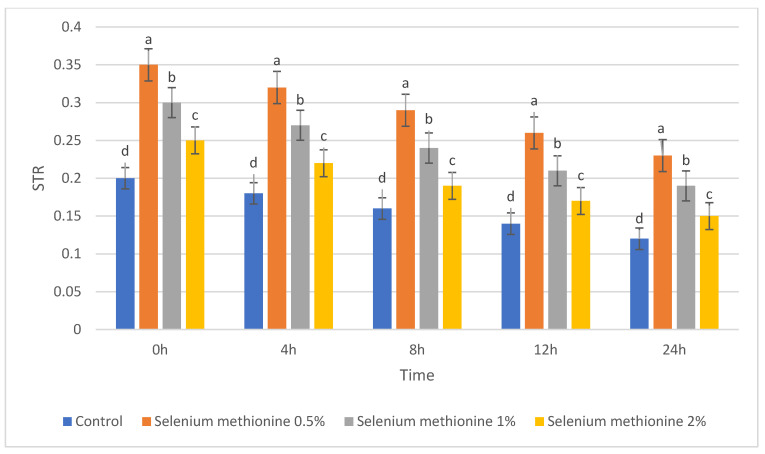
Changes in straightness index (STR) of rooster sperm during storage at 25 °C following selenium methionine supplementation. Data are presented as mean ± SEM (*n* = 3 experimental replicates). Different superscript letters indicate significant differences among treatments at the same time point (*p* < 0.05).

For clarity, each semen quality parameter is presented in a separate table and figure to allow independent visualization of treatment and storage-time effects. The parameters of semen quality were evaluated during storage at 25 °C after supplementation with selenium methionine (SM) at concentrations of 0.5%, 1%, and 2%. These parameters included total sperm motility, dead sperm percentage, sperm viability, and kinematic parameters (VSL, VCL, VAP, ALH, LIN, and STR). The corresponding results are presented in Table 1, Table 2, Table 3, Table 4, Table 5, Table 6, Table 7, Table 8 and Table 9 and Figure 1, Figure 2, Figure 3, Figure 4, Figure 5, Figure 6, Figure 7, Figure 8 and Figure 9. Each parameter is described in detail in the following subsections. All groups showed time-dependent decreases during storage, consistent with previous reports [38,56,57].

Statistical analysis revealed significant effects of treatment, storage time, and their interaction (treatment × time) on most semen quality parameters (*p* < 0.05).

#### 3.1.1. Total Sperm Motility (%)

In the present study, sperm survivability was assessed using total sperm motility as the primary functional indicator during storage. The results for total sperm motility are presented in Table 1 and Figure 1. Total sperm motility gradually decreased over the 24 h storage period in all groups.

At 0 h, selenium methionine 0.5% showed significantly higher motility (90.00%) than Control (70.00%), while selenium methionine 1% and selenium methionine 2% showed intermediate values (*p* < 0.05). After 4 h, a distinct concentration-dependent trend was noticed: selenium methionine 0.5% > selenium methionine 1% > selenium methionine 2% > Control. At 24 h, total sperm motility was highest in the selenium methionine 0.5% (70.00%), followed by selenium methionine 1% (65.00%) and selenium methionine 2% (60.00%), whereas Control showed a substantial decrease (40.00%). These results indicate that 0.5% selenium methionine provided the greatest protective effect among the tested concentrations during ambient storage.

#### 3.1.2. Dead Sperm Percentage (%)

The results for dead sperm percentage are presented in Table 2 and Figure 2. Dead sperm percentage increased progressively during storage in all groups. However, selenium methionine supplementation significantly reduced dead sperm percentage compared with the control group at all evaluated time points (*p* < 0.05).

Dead sperm percentage was determined as the proportion of non-motile spermatozoa detected by the CASA system and was therefore interpreted as the inverse functional indicator of total sperm motility during storage rather than being mathematically calculated as the complement of survivability.

Among the tested concentrations, the selenium methionine 0.5% group consistently showed the lowest dead sperm percentage, followed by selenium methionine 1% and selenium methionine 2%. At 24 h, dead sperm percentage in the selenium methionine 0.5% group (30.00%) was approximately half of that observed in the control group (60.00%). These findings indicate that selenium methionine supplementation reduced sperm deterioration during ambient storage, with the strongest protective effect observed at 0.5%.

#### 3.1.3. Sperm Viability (%)

The results for sperm viability are presented in Table 3 and Figure 3. Sperm viability decreased progressively over time in all groups during storage at 25 °C. Nevertheless, selenium methionine supplementation significantly preserved sperm viability compared with the control group at all evaluated time points (*p* < 0.05).

Among the tested concentrations, selenium methionine 0.5% maintained the highest sperm viability throughout storage, followed by selenium methionine 1% and selenium methionine 2%. At 24 h, sperm viability was 50.00% in the selenium methionine 0.5% group, whereas the control group declined to 20.00%. These findings further support the superior protective effect of 0.5% selenium methionine among the tested concentrations.

#### 3.1.4. Kinematic Parameters (VSL, VCL, and VAP)

The results for the kinematic parameters VSL, VCL, and VAP are presented in Table 4, Table 5 and Table 6 and Figure 4, Figure 5 and Figure 6. All three velocity parameters declined progressively during storage in all groups. However, selenium methionine supplementation significantly preserved these kinematic values compared with the control group, with the strongest effect consistently observed in the selenium methionine 0.5% group.

The results for VSL are presented in Table 4 and Figure 4. At all storage times, VSL values followed the order selenium methionine 0.5% > selenium methionine 1% > selenium methionine 2% > Control. The selenium methionine 0.5% group maintained the highest VSL values from 0 h to 24 h, indicating better preservation of sperm movement quality during storage [50,58].

The results for VCL are presented in Table 5 and Figure 5. A similar time-dependent decline was observed in all groups; however, selenium methionine supplementation significantly improved VCL compared with the control group. The selenium methionine 0.5% group consistently showed the highest VCL values throughout storage.

The results for VAP are presented in Table 6 and Figure 6. VAP values decreased with increasing storage time in all groups, but selenium methionine-treated samples maintained higher values than the control. Again, the selenium methionine 0.5% group showed the greatest preservation at all evaluated time points.

#### 3.1.5. ALH, LIN, and STR

The results for ALH, LIN, and STR are presented in Table 7, Table 8 and Table 9 and Figure 7, Figure 8 and Figure 9. These parameters also declined progressively during storage in all groups. However, selenium methionine supplementation preserved higher values than the control group, with the greatest protective effect, again, observed in the selenium methionine 0.5% treatment [38,56].

The results for ALH are presented in Table 7 and Figure 7. ALH decreased over time in all groups, but the decline was less marked in selenium methionine-supplemented samples, especially in the selenium methionine 0.5% group.

The results for LIN are presented in Table 8 and Figure 8. LIN values declined progressively during storage, but selenium methionine supplementation maintained significantly higher values than the control group at several time points.

The results for STR are presented in Table 9 and Figure 9. As with the other kinematic indicators, STR decreased during storage in all groups, but selenium methionine 0.5% consistently showed the highest values among the tested concentrations.

Across all storage intervals, a consistent hierarchy was observed (selenium methionine 0.5% > selenium methionine 1% > selenium methionine 2% > Control), with significant treatment effects at each time point (*p* < 0.05).

### 3.2. Dose–Response (Polynomial) Analysis at 25 °C

To gain a better insight into the dose–response relationships, a polynomial contrast analysis (linear, quadratic, and cubic terms) was performed to examine the relationship between selenium methionine concentration and semen quality parameters during storage at 25 °C. Polynomial modeling has been widely used in biological dose–response studies where responses are not linear but rather threshold, biphasic, or hormetic. Non-linear regression models can be used to better describe complex biological responses than linear models [53,59]. In most parameters tested, linear terms were not significant (*p* > 0.05), while quadratic and cubic terms showed better goodness-of-fit statistics. The preponderance of higher-order polynomial terms indicates that the biological effect of selenium methionine is multi-phasic rather than dose-proportional [5,11,60].

#### 3.2.1. Total Sperm Motility

**Figure 10:**
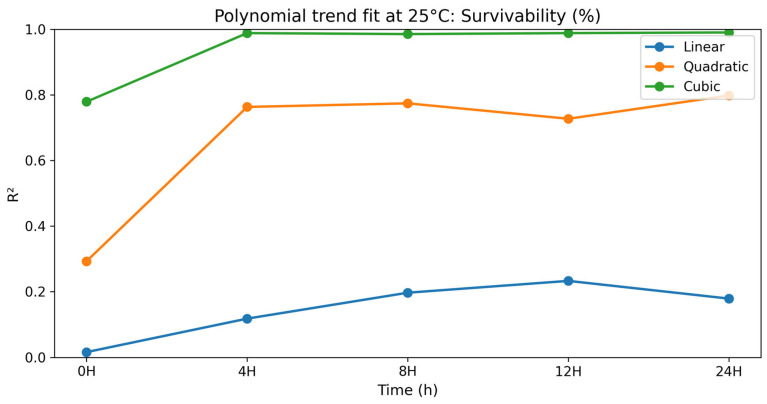
Polynomial trend fit (R^2^) over storage time at 25 °C for total sperm motility (%).

The polynomial contrast results for total sperm motility are presented in Table 10 and Figure 10. For total sperm motility, the cubic component was significant at 0 h (R^2^ = 0.779; *p* = 0.005), with the linear (*p* = 0.694) and quadratic (*p* = 0.210) components being insignificant. From 4 h to 24 h, the cubic components were highly significant (R^2^ = 0.985–0.990; *p* < 0.0001), with significant quadratic components from 4 h onwards (*p* = 0.001–0.003). Throughout the storage period, total sperm motility was always in the order of selenium methionine 0.5% > selenium methionine 1% > selenium methionine 2%, indicating that increasing the concentration of selenium methionine beyond 0.5% did not enhance total sperm motility during storage at 25 °C. These results indicate a significant non-linear dose–response relationship between selenium methionine concentration and sperm motility, with the cubic model providing the best statistical fit and demonstrating that 0.5% selenium methionine maintained the highest motility throughout the storage period.

#### 3.2.2. Sperm Mortality

**Figure 11:**
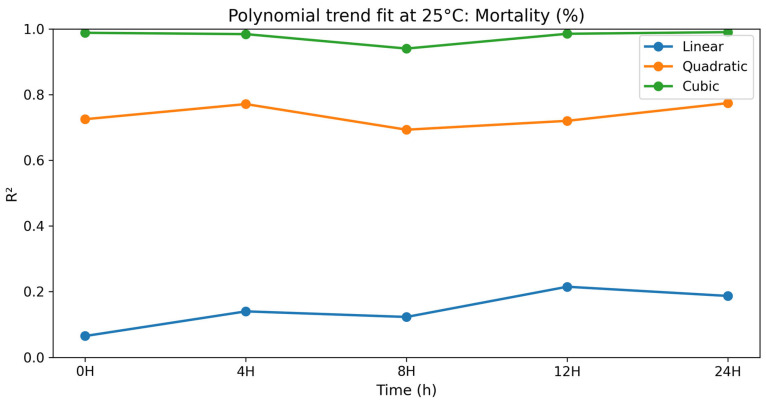
Polynomial trend fit (R^2^) over storage time at 25 °C for mortality (%).

The polynomial contrast results for sperm mortality are presented in Table 11 and Figure 11. For sperm mortality, linear trends were not significant at any point in time (*p* = 0.129–0.423), suggesting the lack of a linear dose–response with increasing selenium methionine concentration. The quadratic terms were significant at all time points (*p* = 0.001–0.005). The cubic term had the best fit for the duration of storage (R^2^ = 0.940–0.990; *p* < 0.0001). Mortality was lowest in the selenium methionine 0.5% group at all points in time, with progressively increasing values in the selenium methionine 1% and selenium methionine 2% groups, indicating a non-linear dose–response relationship between selenium methionine concentration and sperm mortality during storage at 25 °C. The significant quadratic and cubic components indicate that sperm mortality responded to selenium methionine supplementation in a non-linear manner, with 0.5% selenium methionine consistently minimizing sperm mortality across all storage intervals.

#### 3.2.3. Sperm Viability

**Figure 12:**
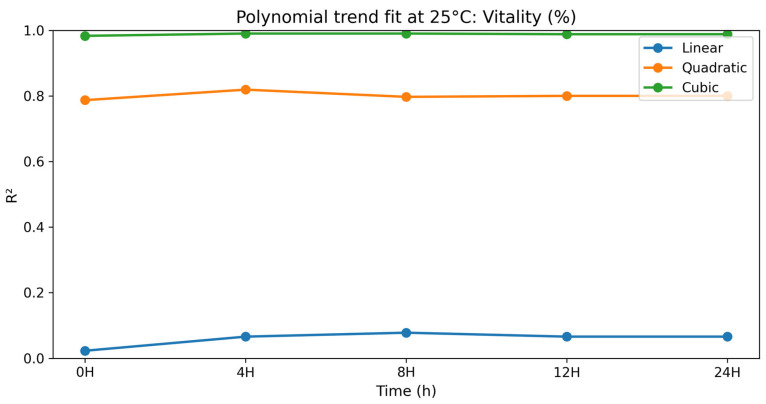
Polynomial trend fit (R^2^) over storage time at 25 °C for sperm viability (%).

The polynomial contrast results for sperm viability are presented in Table 12 and Figure 12. For sperm viability, linear terms were not significant at any time point (*p* = 0.379–0.636). The quadratic and cubic terms were significant at all time points, and the cubic terms had excellent fit values (R^2^ = 0.983–0.990) with *p* < 0.0001. Sperm viability was highest in selenium methionine 0.5% at all times, followed by selenium methionine 1% and selenium methionine 2%, indicating that the optimal dose–response was achieved at 0.5% selenium methionine under 25 °C conditions. The strong significance of the cubic models together with high goodness-of-fit values indicates that selenium methionine supplementation exerted a significant non-linear protective effect on sperm viability, with 0.5% selenium methionine providing the most stable viability during storage at 25 °C.

#### 3.2.4. Straight-Line Velocity (VSL)

**Figure 13:**
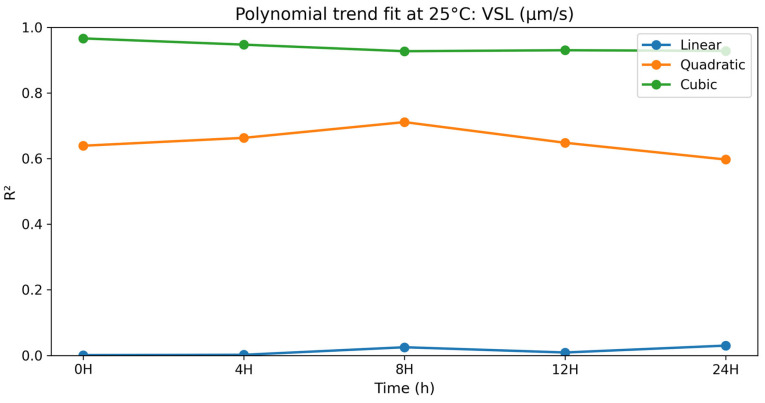
Polynomial trend fit (R^2^) over storage time at 25 °C for VSL (μm/s).

The polynomial contrast results for straight-line velocity are presented in Table 13 and Figure 13. For VSL, linear terms were not significant over the storage period (*p* = 0.591–0.939), while quadratic terms were significant at all points (*p* = 0.004–0.017), and cubic terms showed the best fit at all points (R^2^ = 0.927–0.966; *p* < 0.0001). At all points, VSL was maximum in selenium methionine 0.5%, followed by selenium methionine 1% and selenium methionine 2%, indicating a non-linear dose–response pattern, with kinematic performance declining at higher selenium methionine concentrations. These results indicate that straight-line velocity exhibited a non-linear response to selenium methionine supplementation, with the cubic model showing the best fit and 0.5% selenium methionine maintaining the highest velocity values throughout the storage period.

#### 3.2.5. Curvilinear Velocity (VCL)

**Figure 14:**
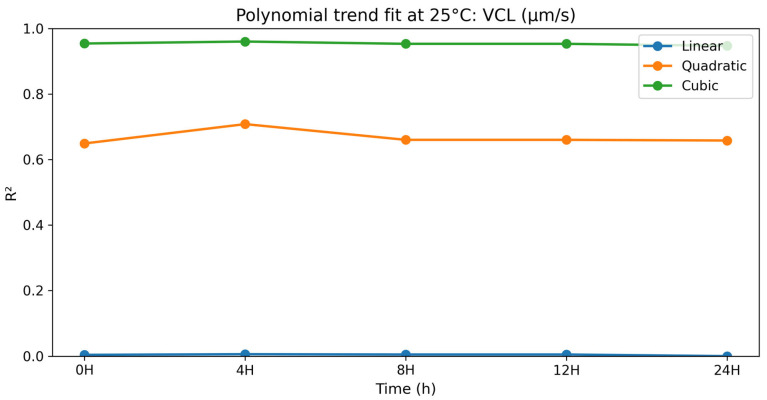
Polynomial trend fit (R^2^) over storage time at 25 °C for VCL (μm/s).

The polynomial contrast results for curvilinear velocity are presented in Table 14 and Figure 14. For VCL, linear components were non-significant at all time points (*p* = 0.810–0.948). Quadratic trends were significant at all time points (*p* = 0.004–0.009), and cubic components had very high fit values (R^2^ = 0.947–0.960; *p* < 0.0001). The values of VCL were consistently higher in the order selenium methionine 0.5% > selenium methionine 1% > selenium methionine 2%, indicating that the optimal kinematic response at 25 °C occurred at 0.5% selenium methionine. The strong cubic model fit suggests that curvilinear velocity followed a multi-phasic dose–response pattern, with the optimal kinematic performance consistently observed at 0.5% selenium methionine.

#### 3.2.6. Average Path Velocity (VAP)

**Figure 15:**
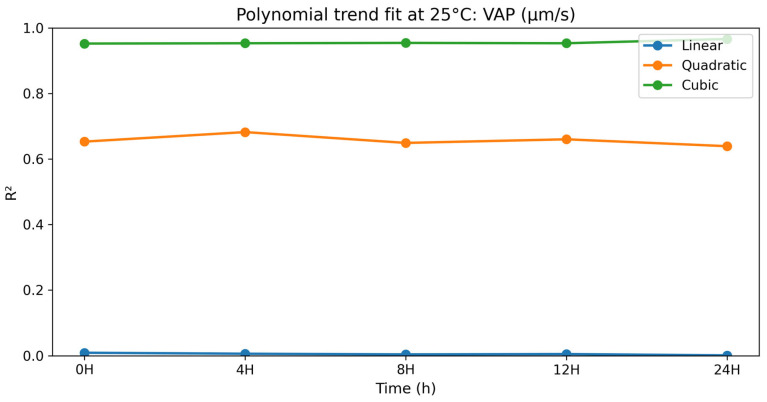
Polynomial trend fit (R^2^) over storage time at 25 °C for VAP (μm/s).

The polynomial contrast results for average path velocity are presented in Table 15 and Figure 15. For VAP, the linear trends were not significant at the various time points (*p* = 0.765–0.939). The quadratic terms were significant at all time points (*p* = 0.006–0.010), and the best fit was consistently obtained by the cubic models (R^2^ = 0.952–0.966; *p* < 0.0001). VAP was consistently highest at selenium methionine 0.5%, followed by selenium methionine 1% and selenium methionine 2% at all stages of storage, indicating a consistent non-linear dose–response relationship at 25 °C storage conditions. These findings further confirm that the response of average path velocity to selenium methionine supplementation followed a non-linear dose–response pattern.

#### 3.2.7. Amplitude of Lateral Head Displacement (ALH)

**Figure 16:**
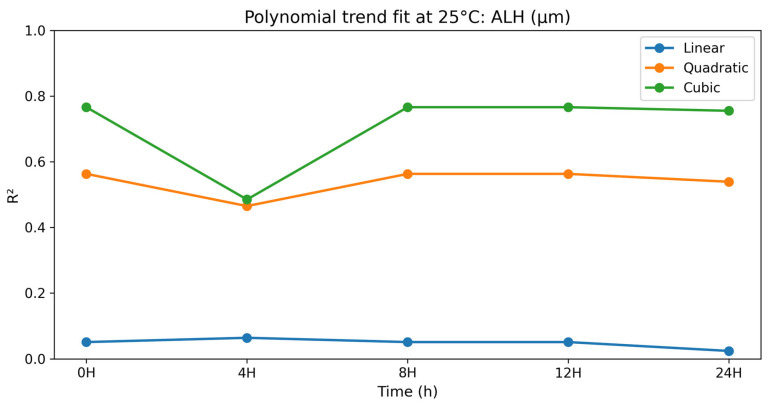
Polynomial trend fit (R^2^) over storage time at 25 °C for ALH (μm).

The polynomial contrast results for the amplitude of lateral head displacement are presented in Table 16 and Figure 16. For ALH, the cubic term was significant at 0 h (*p* = 0.007), 8 h (*p* = 0.007), 12 h (*p* = 0.007), and 24 h (*p* = 0.008) with R^2^ values of 0.755 to 0.766. However, at 4 h, the trend terms were not as strong and failed to achieve significance for the cubic term (*p* = 0.132). In general, the ALH was higher at selenium methionine 0.5% than at the higher concentrations, indicating a non-linear response to increasing selenium methionine concentrations. The polynomial analysis suggests that ALH responses to selenium methionine supplementation were moderately non-linear, with 0.5% selenium methionine generally maintaining higher head displacement amplitudes compared with higher concentrations.

#### 3.2.8. Linearity (LIN)

**Figure 17:**
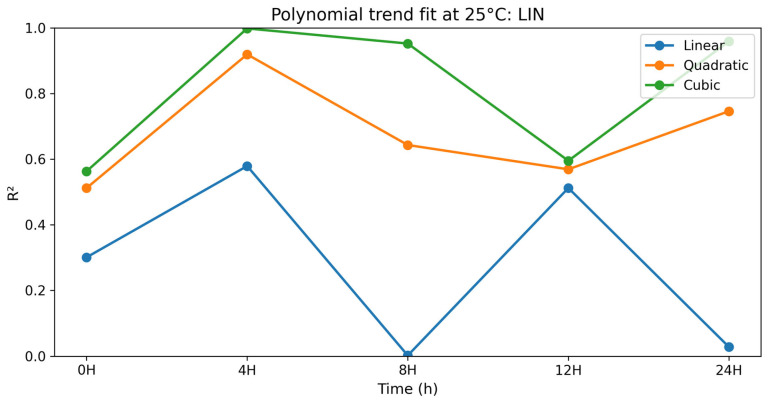
Polynomial trend fit (R^2^) over storage time at 25 °C for LIN.

The polynomial contrast results for linearity are presented in Table 17 and Figure 17. For LIN, the time-dependent variation in the polynomial terms was noted. At 0 h, the quadratic term was significant (*p* = 0.040), but the cubic term was not significant (*p* = 0.072). At 4 h, the linear, quadratic, and cubic terms were all significant (linear *p* = 0.004; quadratic *p* < 0.0001; cubic *p* < 0.0001), with the cubic model having a near-perfect fit (R^2^ = 0.998). At 8 h and 24 h, the cubic terms were highly significant (*p* < 0.0001). In all cases, the LIN was highest in selenium methionine 0.5%, followed by selenium methionine 1% and selenium methionine 2%, indicating a non-linear dose–response relationship between selenium methionine concentration and sperm trajectory linearity. The significant higher-order polynomial terms indicate that LIN exhibited a complex dose–response relationship, with 0.5% selenium methionine consistently providing the most favorable linearity values during storage.

#### 3.2.9. Straightness (STR)

**Figure 18:**
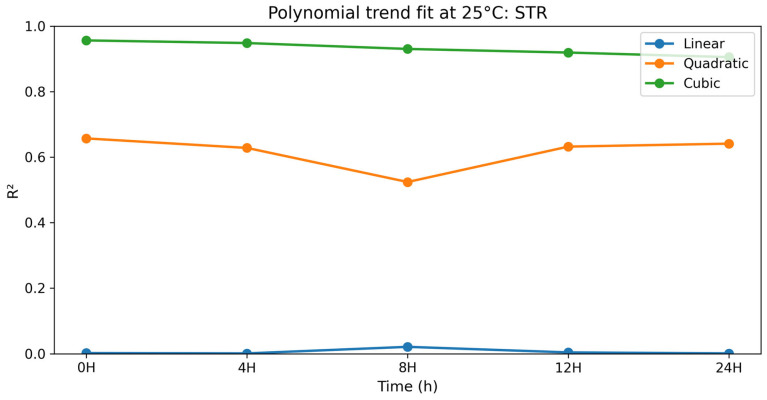
Polynomial trend fit (R^2^) over storage time at 25 °C for STR.

The polynomial contrast results for straightness are presented in Table 18 and Figure 18. For STR, linear terms were not significant at any time point (*p* = 0.651–0.917), while quadratic terms were significant at all time points (*p* = 0.008–0.035). Cubic terms showed excellent fit values (R^2^ = 0.905–0.956) and were significant at all time points (*p* < 0.0001). STR was always highest at selenium methionine 0.5% and followed by selenium methionine 1% and selenium methionine 2%, which revealed that beyond selenium methionine 0.5%, further increase in selenium methionine concentration did not result in any improvement in STR. The strong cubic trend and significant quadratic components indicate that the straightness index responded non-linearly to selenium methionine supplementation, with 0.5% selenium methionine producing the most stable STR values across storage time.

### 3.3. Effect of Selenium Methionine on Acrosome Integrity at 25 °C

**Figure 19:**
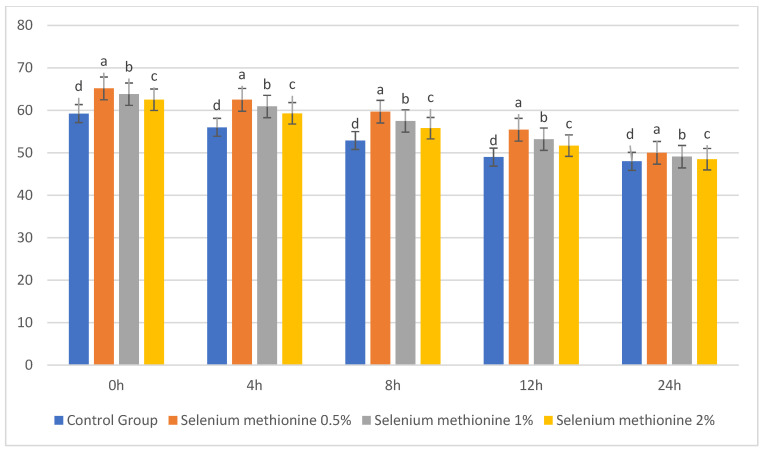
Changes in acrosome integrity (%) of rooster sperm during storage at 25 °C following SM supplementation. Data are presented as mean ± SEM (*n* = 3 experimental replicates). Different superscripts indicate significant differences among treatments at the same storage time (*p* < 0.05).

The results for acrosome integrity are presented in Table 19 and Figure 19. The integrity of the acrosome was progressively reduced during storage in all groups. However, selenium methionine supplementation resulted in a significant maintenance of acrosomal membrane integrity compared to Control (*p* < 0.05). In the early time points (0 h and 4 h), the highest values of acrosome integrity were found in selenium methionine 0.5%. Although all groups showed a reduction in integrity at 24 h, the selenium methionine groups showed better maintenance of structural integrity compared to Control. These results indicate that selenium methionine could protect the acrosomal membranes by reducing oxidative lipid peroxidation during storage at 25 °C. The superiority of selenium methionine 0.5% at all time points indicates optimal antioxidant preservation of acrosomal membranes without causing redox imbalance at higher concentrations [61].

### 3.4. Effect of Selenium Methionine on Plasma Membrane Integrity at 25 °C

**Figure 20:**
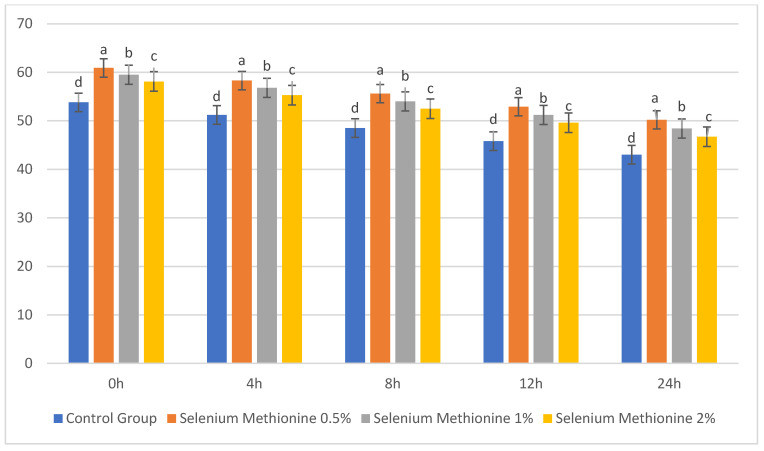
Changes in sperm plasma membrane integrity (%) during storage at 25 °C following selenium methionine supplementation. Values are expressed as mean ± SEM (*n* = 3 experimental replicates). Different superscripts indicate significant differences among treatments within the same time interval (*p* < 0.05).

The results for plasma membrane integrity are presented in Table 20 and Figure 20. The integrity of the sperm plasma membrane was compromised with increasing storage time in all groups. However, the supplementation of selenium methionine significantly improved the membrane integrity compared to the control group. Selenium methionine 0.5% showed the highest membrane integrity at all times, with the lowest rate of decline between 0 h and 24 h. This finding suggests better stabilization of the membrane and lower oxidative damage in selenium-supplemented groups [62,63]. The protective action was concentration-dependent but showed diminishing returns at higher concentrations.

At 0 h, the highest integrity of the plasma membrane was found in selenium methionine 0.5% (60.90 ± 0.64%), followed by selenium methionine 1% (59.50 ± 0.83%) and selenium methionine 2% (58.10 ± 0.73%), which were significantly higher than the control group (53.80 ± 0.39%).

At 4 h and 8 h, the selenium methionine-supplemented groups showed significantly higher membrane integrity than the control group (*p* < 0.05). The highest integrity was found in the selenium methionine 0.5% group.

At 12 h and 24 h, the membrane integrity was reduced in all groups; however, selenium methionine 0.5% was still relatively superior to selenium methionine 1%, selenium methionine 2%, and control.

At 24 h of storage, the percentage reduction from baseline was least in selenium methionine 0.5% (17.6%), followed by selenium methionine 1% (18.7%), selenium methionine 2% (19.6%), and control (20.1%).

**Figure 21:**
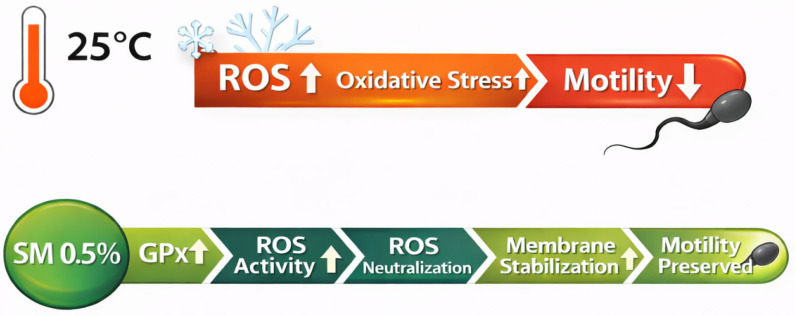
Schematic illustration of the proposed mechanism by which selenium methionine (SM) supplementation protects rooster sperm during storage at 25 °C. Storage at ambient temperature increases reactive oxygen species (ROS), leading to oxidative stress and reduced sperm motility. Selenium methionine supplementation enhances antioxidant defense through increased glutathione peroxidase (GPx) activity, promoting ROS neutralization, stabilizing the sperm plasma membrane, and ultimately preserving sperm motility.

The results clearly show that selenium methionine supplementation reduced the deterioration of the membrane. The proposed protective mechanism by which selenium methionine mitigates oxidative stress and stabilizes the sperm plasma membrane is illustrated in Figure 21.

### 3.5. Correlation Analysis Among Functional, Kinematic, and Structural Parameters at 25 °C

The Pearson correlation results are presented in Table 21. To assess the inter-relationships between functional (total sperm motility, mortality, and sperm viability), kinematic (VSL, VCL, VAP, ALH, LIN, and STR), and structural (acrosome and plasma membrane integrity) variables under storage conditions at 25 °C, Pearson correlation analysis was carried out using 20 observations (4 treatments × 5 time points).

Total sperm motility and sperm viability showed strong positive correlations with velocity variables (VSL, VCL, and VAP) and structural integrity indices (acrosome and plasma membrane integrity) (r = 0.82–0.98, *p* < 0.001) [64,65]. However, mortality showed strong negative correlations with these variables (r = −0.80 to −0.97, *p* < 0.001). Plasma membrane integrity showed strong positive correlations with motility variables, especially VSL and VAP, suggesting that the maintenance of membrane stability was strongly associated with the maintenance of progressive motility.

Acrosome integrity showed moderate to strong correlations with both kinematic and sperm viability variables (r = 0.65–0.91, *p* < 0.01), thus validating its use as a marker for structural preservation during ambient storage conditions [66].

The pattern of correlation confirms the internal consistency of the data on biological principles: those variables that maintained integrity also maintained motility, while variables that indicated deterioration (mortality) had inverse correlations.

Since correlations were calculated from group means, common trends with time may increase the value of correlation coefficients. Hence, the data are interpreted as indicative of association rather than causation [55]. Statistical significance was considered at *p* < 0.05.

### 3.6. Flow Cytometric Evaluation of Sperm Physiological Status at 25 °C

#### 3.6.1. Representative Dot Plot Distribution

**Figure 22:**
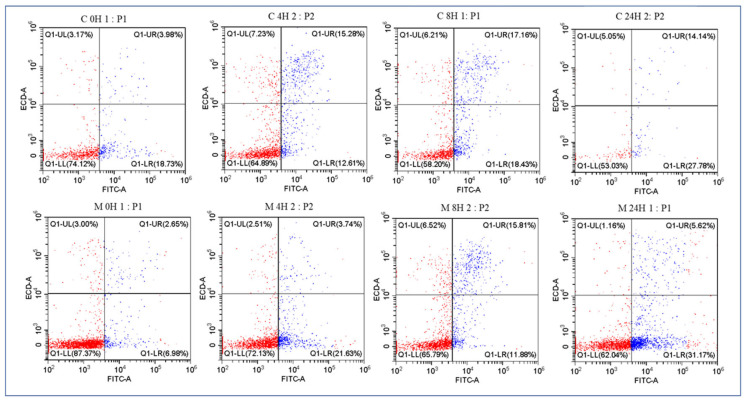
Representative flow cytometric dot plots showing sperm population distribution (viable, early apoptotic, late apoptotic, and necrotic cells) in control and selenium methionine 0.5% (favorable concentration) groups during storage at 25 °C.

Representative flow cytometric dot plots are presented in Figure 22. To better understand the physiological condition of spermatozoa, Annexin V-FITC/PI staining was conducted [48,67,68]. Representative dot plots are shown for Control and selenium methionine 0.5% (favorable concentration) groups during storage at 25 °C. Dot plot analysis demonstrated a time-dependent transition from viable to apoptotic and necrotic populations. The selenium methionine 0.5% group maintained a higher proportion of viable sperm compared to Control, particularly at early storage intervals (0 h and 4 h).

At 0 h, the Control showed 74.12% viable sperm (Q1-LL), with early apoptotic (18.73%), late apoptotic (3.17%), and necrotic cells (3.98%). In contrast, the selenium methionine-treated group showed significantly higher sperm viability (87.37%) and lower early apoptotic cells (6.98%), reflecting an immediate protective stabilization of membrane integrity.

At 4 h, sperm viability decreased in both groups; however, selenium methionine maintained a higher percentage of viable sperm (72.13%) compared to Control (64.89%). Apoptotic progression was more advanced in Control, as reflected by higher late apoptotic and necrotic cell percentages.

At 8 h, the protective effect of selenium methionine continued, with 65.79% viable sperm compared to 58.20% in Control. Although early apoptosis increased with time, selenium methionine consistently suppressed excessive necrotic transformation.

At 24 h, a substantial reduction in overall sperm viability was noted; however, selenium methionine maintained 62.04% viable sperm compared to 53.03% in Control. Notably, necrotic cell accumulation was lower in selenium methionine-treated samples, suggesting attenuation of irreversible membrane damage.

In summary, the dot plot distribution patterns reveal that selenium methionine supplementation delayed apoptotic progression and suppressed necrotic transformation during ambient storage.

#### 3.6.2. Quantitative Analysis of Viable and Apoptotic Populations

**Figure 23:**
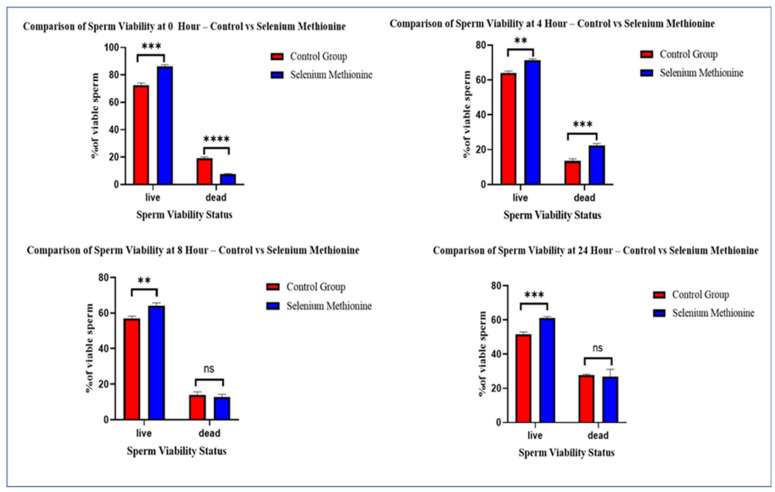
Quantitative analysis of sperm physiological status determined by flow cytometry during storage at 25 °C. Bars represent percentages of viable, early apoptotic, late apoptotic, and necrotic sperm populations (mean ± SD). Asterisks indicate significant differences between control and selenium methionine groups (** *p* < 0.01, *** *p* < 0.001, **** *p* < 0.0001, ns “not significant” *p* ≥ 0.05).

The quantitative flow cytometry results are presented in Figure 23. Quantitative analysis revealed a significantly greater percentage of viable sperm in selenium methionine-treated groups than in the control group at 0 h (*p* < 0.001), 4 h (*p* < 0.01), 8 h (*p* < 0.01), and 24 h (*p* < 0.001). Despite the reduction in sperm viability, selenium supplementation retarded the apoptotic process and decreased necrotic change [63].

At 0 h, selenium methionine caused a significant increase in the percentage of viable sperm (*p* < 0.001) and a decrease in dead sperm percentage compared to Control.

At 4 h, the sperm viability was significantly higher in selenium methionine-treated samples (*p* < 0.01), showing continued cytoprotective effects.

At 8 h, sperm viability was still higher in selenium methionine-treated samples (*p* < 0.01), although the apoptotic process was more advanced in both samples, as expected due to time-dependent physiological changes.

At 24 h, selenium methionine-treated sperm had a significantly higher sperm viability (*p* < 0.001) than Control. Although deterioration was observed at room temperature, selenium methionine prevented excessive necrotic transformation.

In summary, flow cytometry results show that selenium methionine effectively protected sperm membranes and retarded the apoptotic process during short-term storage at 25 °C.

## 4. Discussion

The present study demonstrates that selenium methionine (SM) supplementation in rooster semen extender significantly preserved functional, kinematic, and structural parameters of semen quality during liquid storage at 25 °C. As anticipated under ambient storage conditions, all indices of semen quality were observed to decrease over time, indicating a progressive loss of sperm physiological function during in vitro storage [38,56,57]. However, the extent of this progressive loss was significantly reduced by selenium methionine supplementation, with the most consistent protective effect being observed at 0.5%.

Total sperm motility and sperm viability progressively declined over time, while mortality increased reciprocally. This time-dependent pattern of sperm damage agrees with the suggestion that spermatozoa, lacking cytoplasmic antioxidant mechanisms and being vulnerable to oxidative damage of their membranes, undergo progressive damage during liquid storage [5,11]. The substantial increase in total sperm motility and decrease in mortality in the selenium-supplemented groups clearly establishes the efficacy of selenium methionine in protecting spermatozoa from oxidative damage during storage. The higher efficacy of the 0.5% group indicates that the protective action of selenium is not dose-proportion-dependent and that higher doses become counterproductive.

The CASA-derived parameters for velocity, namely, VSL, VCL, and VAP, showed the typical pattern of concentration-dependent preservation, where the profile showed the trend of selenium methionine 0.5% > selenium methionine 1% > selenium methionine 2% > Control. The parameters for progressive motility and velocities are indicative of the activity of mitochondrial ATP production and flagellar movement, respectively, which are extremely sensitive to the accumulation of ROS and the peroxidation of cellular membranes during storage [50,58]. The sustained velocities in the 0.5% group are in line with the hypothesis that the favorable concentration of selenium maintains the integrity of the mitochondria and the energy production necessary for the process of progressive motility.

Selenium plays a key biochemical role in mitochondrial protection through its incorporation into several selenoproteins, particularly glutathione peroxidase (GPx) isoforms such as GPx1 and GPx4. These enzymes catalyze the reduction of hydrogen peroxide and lipid hydroperoxides, thereby limiting oxidative damage to mitochondrial membranes and preserving mitochondrial membrane potential. Maintenance of mitochondrial integrity is essential for ATP production required for flagellar beating and progressive sperm motility. Therefore, the improved kinematic parameters observed in the selenium methionine-supplemented groups may be associated with enhanced antioxidant enzyme activity and stabilization of mitochondrial function, which together reduce ROS-induced mitochondrial dysfunction during liquid storage.

The parameters for trajectory, namely, LIN and STR, and the parameter for amplitude of lateral head movement, namely, ALH, showed the expected trend of reduced values with time due to the reduced coordination of sperm movement during stress conditions. However, the higher values of LIN and STR in the 0.5% selenium methionine group, especially at the later time points, are indicative of the better preservation of the stability of the movement of the sperm.

The integrity of the acrosome and plasma membrane is essential for sperm survival and fertilizing capacity. It has been observed that the integrity of the membrane and the acrosome decreases with time when stored at 25 °C. However, selenium methionine preserved membrane integrity, and 0.5% showed the most favorable protective effect among the tested concentrations. The integrity of the acrosomal membrane has been observed to be highly sensitive to oxidative stress caused by lipid peroxidation, which affects its integrity [61]. Moreover, the preservation of the integrity of the plasma membrane implies a decrease in oxidative lipid damage [62], which is due to the decrease in the rate of degeneration in the 0.5% group.

A significant advantage of the research is that the polynomial contrast analysis was carried out, which showed that the effect of selenium methionine was non-linear, with quadratic and cubic effects being the most appropriate in fitting the results for most parameters. This is in agreement with the hormesis theory in that while low levels of antioxidants are desirable, higher levels can actually be detrimental to the body [5,60]. This is the reason why an increase in the percentage of the supplement from 0.5% to 1% or 2% does not necessarily increase the results in a proportional manner, but can actually decrease them.

Pearson correlation analysis also provided further evidence of biological validity. Functional variables (total sperm motility and sperm viability) were strongly positively correlated with velocity variables and membrane/acrosome integrity, while mortality was strongly negatively correlated. This is in keeping with a mechanistic model in that maintenance of membrane integrity is associated with maintenance of motility and reduced cell death. Again, as mentioned, this is correlation and calculation is performed on group means; a common trend over time may increase correlation coefficients; thus, this should be considered in relation to association rather than causation [55]. Partial correlation analysis, adjusting for storage time, could also be considered in future research.

The results obtained from Annexin V-FITC/PI staining showed that the selenium methionine supplementation delayed the onset of the process of apoptosis/necrosis during storage. Annexin V is known to be associated with the early stages of apoptosis due to the externalization of phosphatidylserine. Positive staining with PI is associated with membrane damage [48,67,68]. The increase in the percentage of viable cells in the selenium methionine-supplemented samples compared to the controls is in agreement with the proposed mechanism that selenium methionine stabilizes the architecture of the cell membrane to prevent oxidative membrane damage [63].

From the application perspective, the ambient storage at 25 °C is relevant for the semen handling and transportation in most practical AI schemes where refrigeration facilities are not easily accessible. The present findings indicate that direct supplementation of selenium methionine with a concentration of 0.5%, which produced the most consistent protective effects among the tested concentrations, could substantially improve semen quality in terms of functional, kinematic, and structural parameters during the 24 h storage period.

Although the present study focused on semen quality parameters during liquid storage, the practical value of these improvements ultimately depends on their impact on reproductive performance. Therefore, future studies should investigate whether the enhanced motility, membrane integrity, and reduced apoptotic progression observed with selenium methionine supplementation translate into improved fertility and hatchability outcomes in artificial insemination programs. Such studies would further validate the practical application of selenium methionine supplementation in commercial poultry breeding systems.

The present study has the limitation that the semen samples were collected during a single sampling session. Though the semen samples were pooled, which reduces the variability among males and allows comparison among treatments, it is recommended that the present findings be validated on more than one day of semen collection.

**Figure 24:**
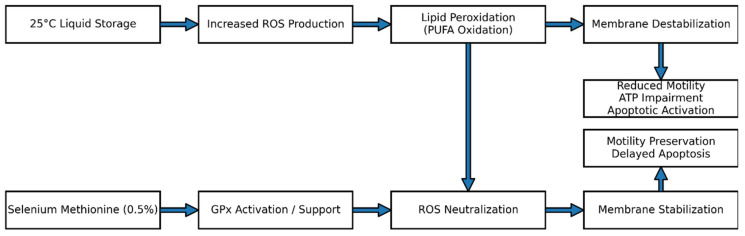
Proposed mechanistic model illustrating the protective effects of selenium methionine (0.5%) during rooster semen storage at 25 °C. Ambient storage increases reactive oxygen species (ROS) production, inducing lipid peroxidation, membrane destabilization, motility impairment, and apoptotic activation. Most favorable selenium methionine concentration enhances glutathione peroxidase (GPx)-mediated antioxidant defense, promotes ROS neutralization, stabilizes sperm membranes, preserves motility, and delays apoptosis.

Based on the integrated functional, structural, and physiological findings of this study, a model is proposed to explain the protective role of selenium methionine during liquid storage at 25 °C (Figure 24). Storage at room temperature may promote continued mitochondrial respiration with the production of ROS, which would induce lipid peroxidation of polyunsaturated fatty acids in the membrane, leading to motility inhibition, ATP depletion, and activation of apoptosis. Supplementation with selenium methionine at 0.5%, which showed the most favorable effects among the tested concentrations, may increase glutathione peroxidase activity, thus neutralizing ROS, maintaining membrane integrity, motility parameters, and inhibiting apoptosis.

## 5. Future Directions and Research Needs

However, the results suggest that the use of 0.5% selenium methionine provides consistent protection during the storage period at 25 °C. Nevertheless, there are some aspects that need further research.

Firstly, it is important to confirm if fertility and hatchability rates are improved in artificial insemination programs. Although semen characteristics in vitro are important, they become more significant in a real-world scenario.

Secondly, further research is required to study the antioxidant enzyme activity, mitochondrial function, and lipid peroxidation. More knowledge about the biological mechanisms involved in the protective effects of 0.5% selenium methionine can be achieved.

Second, further research is required to study the antioxidant enzyme activity, mitochondrial function, and lipid peroxidation. More knowledge about the biological mechanisms involved in the protective effects of 0.5% selenium methionine can be achieved.

Third, further research is required to compare the results with other antioxidant compounds. More knowledge about the biological mechanisms involved in the protective effects of 0.5% selenium methionine can be achieved.

Finally, since the margin of safety for the use of selenium is narrow and the dose–response relationship is nonlinear, further research is required to study the concentration thresholds and the long-term redox balance. More knowledge about the biological mechanisms involved in the protective effects of 0.5% selenium methionine can be achieved.

## 6. Conclusions

The addition of selenium methionine (SM) to the rooster semen extender improved the preservation of semen quality during liquid storage at 25 °C. Functional parameters such as total sperm motility and sperm viability were better maintained, while sperm mortality and apoptotic progression were reduced. Structural integrity of the spermatozoa, particularly the acrosome and plasma membrane, was also better preserved in the selenium methionine-supplemented groups.

Among the tested concentrations, 0.5% selenium methionine consistently showed the most favorable protective effects on functional, kinematic, and structural semen parameters during storage. These findings suggest that appropriate antioxidant supplementation can enhance semen stability under ambient storage conditions.

From a practical standpoint, maintaining semen quality during short-term storage at 25 °C is important for artificial insemination (AI) programs, especially in situations where refrigeration facilities are limited. Therefore, supplementation of semen extenders with suitable concentrations of selenium methionine may help improve semen handling and transportation in poultry breeding systems.

However, because concentrations lower than 0.5% were not evaluated in this study, further research is needed to determine the optimal selenium methionine concentration and to assess its effects on fertility and hatchability under practical breeding conditions.

## Figures and Tables

**Table 1:** Effect of selenium methionine supplementation on total sperm motility (%) during storage at 25 °C.

Group	0 h	4 h	8 h	12 h	24 h
Control	70.00 ± 2.08 ^d^	60.00 ± 0.88 ^d^	55.00 ± 0.57 ^d^	50.00 ± 1.20 ^d^	40.00 ± 1.20 ^d^
Selenium methionine 0.5%	90.00 ± 2.08 ^a^	85.00 ± 0.88 ^a^	80.00 ± 1.15 ^a^	75.00 ± 0.57 ^a^	70.00 ± 0.57 ^a^
Selenium methionine 1%	85.00 ± 1.45 ^b^	80.00 ± 1.15 ^b^	75.00 ± 0.88 ^b^	70.00 ± 0.88 ^b^	65.00 ± 1.20 ^b^
Selenium methionine 2%	80.00 ± 1.85 ^c^	75.00 ± 0.88 ^c^	72.00 ± 1.20 ^c^	68.00 ± 1.20 ^c^	60.00 ± 1.45 ^c^

Values are presented as mean ± SEM (*n* = 3). Different superscript letters (a–d) within the same column indicate significant differences among treatment groups at the same time point (*p* < 0.05).

**Table 2:** Effect of selenium methionine supplementation on dead sperm percentage (%) during storage at 25 °C.

Group	0 h	4 h	8 h	12 h	24 h
Control	30.00 ± 0.88 ^d^	40.00 ± 0.88 ^d^	45.00 ± 1.45 ^d^	50.00 ± 1.20 ^d^	60.00 ± 1.17 ^d^
Selenium methionine 0.5%	10.00 ± 0.88 ^a^	15.00 ± 0.57 ^a^	20.00 ± 0.88 ^a^	25.00 ± 0.88 ^a^	30.00 ± 1.15 ^a^
Selenium methionine 1%	15.00 ± 0.88 ^b^	20.00 ± 1.15 ^b^	25.00 ± 0.88 ^b^	30.00 ± 1.20 ^b^	35.00 ± 1.20 ^b^
Selenium methionine 2%	20.00 ± 0.88 ^c^	25.00 ± 0.88 ^c^	28.00 ± 0.88 ^c^	32.00 ± 1.20 ^c^	40.00 ± 1.20 ^c^

Values are presented as mean ± SEM (*n* = 3). Different superscript letters (a–d) within the same column indicate significant differences among treatment groups at the same time point (*p* < 0.05).

**Table 3:** Effect of selenium methionine supplementation on sperm viability (%) during storage at 25 °C.

Group	0 h	4 h	8 h	12 h	24 h
Control	50.00 ± 0.88 ^d^	40.00 ± 1.45 ^d^	35.00 ± 0.88 ^d^	30.00 ± 1.52 ^d^	20.00 ± 0.88 ^d^
Selenium methionine 0.5%	75.00 ± 1.20 ^a^	70.00 ± 0.88 ^a^	65.00 ± 0.57 ^a^	60.00 ± 1.45 ^a^	50.00 ± 1.20 ^a^
Selenium methionine 1%	70.00 ± 1.45 ^b^	65.00 ± 1.15 ^b^	60.00 ± 1.20 ^b^	55.00 ± 0.57 ^b^	45.00 ± 0.88 ^b^
Selenium methionine 2%	60.00 ± 1.45 ^c^	55.00 ± 0.88 ^c^	50.00 ± 1.20 ^c^	45.00 ± 1.20 ^c^	35.00 ± 1.45 ^c^

Values are presented as mean ± SEM (*n* = 3). Different superscript letters (a–d) within the same column indicate significant differences among treatment groups at the same time point (*p* < 0.05).

**Table 4:** Effect of selenium methionine supplementation on straight-line velocity (VSL, µm/s) of rooster sperm during storage at 25 °C.

Group	0 h	4 h	8 h	12 h	24 h
Control	20.00 ± 0.88 ^d^	18.00 ± 1.20 ^d^	16.00 ± 1.15 ^d^	14.00 ± 1.15 ^d^	10.00 ± 0.88 ^d^
Selenium methionine 0.5%	35.00 ± 0.88 ^a^	32.00 ± 0.88 ^a^	28.00 ± 1.45 ^a^	25.00 ± 0.88 ^a^	22.00 ± 0.88 ^a^
Selenium methionine 1%	30.00 ± 0.88 ^b^	27.00 ± 0.88 ^b^	24.00 ± 1.52 ^b^	21.00 ± 1.20 ^b^	18.00 ± 0.57 ^b^
Selenium methionine 2%	25.00 ± 1.15 ^c^	22.00 ± 0.88 ^c^	20.00 ± 1.45 ^c^	18.00 ± 1.20 ^c^	15.00 ± 0.57 ^c^

Values are presented as mean ± SEM (*n* = 3). Different superscript letters (a–d) within the same column indicate significant differences among treatment groups at the same time point (*p* < 0.05).

**Table 5:** Effect of selenium methionine supplementation on curvilinear velocity (VCL, µm/s) of rooster sperm during storage at 25 °C.

Group	0 h	4 h	8 h	12 h	24 h
Control	40.00 ± 1.45 ^d^	35.00 ± 1.52 ^d^	30.00 ± 0.88 ^d^	25.00 ± 0.88 ^d^	20.00 ± 1.20 ^d^
Selenium methionine 0.5%	55.00 ± 1.73 ^a^	50.00 ± 1.45 ^a^	45.00 ± 0.88 ^a^	40.00 ± 0.88 ^a^	35.00 ± 0.88 ^a^
Selenium methionine 1%	50.00 ± 2.33 ^b^	45.00 ± 1.73 ^b^	40.00 ± 0.88 ^b^	35.00 ± 0.88 ^b^	30.00 ± 1.45 ^b^
Selenium methionine 2%	45.00 ± 2.18 ^c^	40.00 ± 1.45 ^c^	35.00 ± 1.45 ^c^	30.00 ± 0.88 ^c^	25.00 ± 0.88 ^c^

Values are presented as mean ± SEM (*n* = 3). Different superscript letters (a–d) within the same column indicate significant differences among treatment groups at the same time point (*p* < 0.05).

**Table 6:** Effect of selenium methionine supplementation on average path velocity (VAP, µm/s) of rooster sperm during storage at 25 °C.

Group	0 h	4 h	8 h	12 h	24 h
Control	30.00 ± 3.28 ^d^	25.00 ± 0.88 ^d^	20.00 ± 0.88 ^d^	15.00 ± 0.88 ^d^	10.00 ± 0.88 ^d^
Selenium methionine 0.5%	45.00 ± 0.88 ^a^	40.00 ± 0.88 ^a^	35.00 ± 0.57 ^a^	30.00 ± 0.88 ^a^	25.00 ± 0.88 ^a^
Selenium methionine 1%	40.00 ± 0.88 ^b^	35.00 ± 1.52 ^b^	30.00 ± 0.57 ^b^	25.00 ± 0.88 ^b^	20.00 ± 0.88 ^b^
Selenium methionine 2%	35.00 ± 0.88 ^c^	30.00 ± 0.88 ^c^	25.00 ± 1.45 ^c^	20.00 ± 0.88 ^c^	15.00 ± 0.88 ^c^

Values are presented as mean ± SEM (*n* = 3). Different superscript letters (a–d) within the same column indicate significant differences among treatment groups at the same time point (*p* < 0.05).

**Table 7:** Effect of selenium methionine supplementation on amplitude of lateral head displacement ALH (µm) of rooster sperm during storage at 25 °C.

Group	0 h	4 h	8 h	12 h	24 h
Control	6.00 ± 0.57 ^d^	5.00 ± 0.88 ^d^	4.00 ± 0.57 ^d^	3.00 ± 0.57 ^d^	2.00 ± 0.28 ^d^
Selenium methionine 0.5%	10.00 ± 0.88 ^a^	9.00 ± 0.57 ^a^	8.00 ± 0.57 ^a^	7.00 ± 0.57 ^a^	6.00 ± 0.57 ^a^
Selenium methionine 1%	9.00 ± 1.20 ^b^	8.00 ± 0.88 ^b^	7.00 ± 0.57 ^b^	6.00 ± 0.88 ^b^	5.00 ± 1.15 ^b^
Selenium methionine 2%	8.00 ± 0.88 ^c^	7.00 ± 0.57 ^c^	6.00 ± 0.57 ^c^	5.00 ± 0.57 ^c^	4.00 ± 0.60 ^c^

Values are presented as mean ± SEM (*n* = 3). Different superscript letters (a–d) within the same column indicate significant differences among treatment groups at the same time point (*p* < 0.05).

**Table 8:** Effect of selenium methionine supplementation on linearity index (LIN) of rooster sperm during storage at 25 °C.

Group	0 h	4 h	8 h	12 h	24 h
Control	0.10 ± 0.01 ^d^	0.08 ± 0.01 ^d^	0.06 ± 0.008 ^d^	0.05 ± 0.008 ^d^	0.04 ± 0.008 ^d^
Selenium methionine 0.5%	0.25 ± 0.008 ^a^	0.22 ± 0.008 ^a^	0.20 ± 0.005 ^a^	0.18 ± 0.008 ^a^	0.16 ± 0.012 ^a^
Selenium methionine 1%	0.20 ± 0.008 ^b^	0.18 ± 0.005 ^b^	0.16 ± 0.011 ^b^	0.15 ± 0.008 ^b^	0.14 ± 0.003 ^b^
Selenium methionine 2%	0.15 ± 0.008 ^c^	0.13 ± 0.005 ^c^	0.11 ± 0.0023 ^c^	0.10 ± 0.005 ^c^	0.09 ± 0.008 ^c^

Values are presented as mean ± SEM (*n* = 3). Different superscript letters (a–d) within the same column indicate significant differences among treatment groups at the same time point (*p* < 0.05).

**Table 9:** Effect of selenium methionine supplementation on straightness index (STR) of rooster sperm during storage at 25 °C.

Group	0 h	4 h	8 h	12 h	24 h
Control	0.20 ± 0.005 ^d^	0.18 ± 0.008 ^d^	0.16 ± 0.008 ^d^	0.14 ± 0.008 ^d^	0.12 ± 0.008 ^d^
Selenium methionine 0.5%	0.35 ± 0.012 ^a^	0.32 ± 0.008 ^a^	0.29 ± 0.005 ^a^	0.26 ± 0.012 ^a^	0.23 ± 0.005 ^a^
Selenium methionine 1%	0.30 ± 0.008 ^b^	0.27 ± 0.005 ^b^	0.24 ± 0.014 ^b^	0.21 ± 0.011 ^b^	0.19 ± 0.005 ^b^
Selenium methionine 2%	0.25 ± 0.008 ^c^	0.22 ± 0.008 ^c^	0.19 ± 0.005 ^c^	0.17 ± 0.012 ^c^	0.15 ± 0.015 ^c^

Values are presented as mean ± SEM (*n* = 3). Different superscript letters (a–d) within the same column indicate significant differences among treatment groups at the same time point (*p* < 0.05).

**Table 10:** Polynomial contrast analysis (linear, quadratic, and cubic components) of selenium methionine supplementation (0, 0.5, 1, and 2%) on total sperm motility (%) during storage at 25 °C.

Time Point	Linear (R^2^, *p*-Value)	Quadratic (R^2^, *p*-Value)	Cubic (R^2^, *p*-Value)
0 h	0.016 (0.694)	0.293 (0.210)	0.779 (0.005)
4 h	0.118 (0.274)	0.763 (0.002)	0.988 (<0.0001)
8 h	0.197 (0.149)	0.774 (0.001)	0.985 (<0.0001)
12 h	0.233 (0.112)	0.727 (0.003)	0.988 (<0.0001)
24 h	0.179 (0.170)	0.798 (0.001)	0.990 (<0.0001)

**Table 11:** Polynomial contrast analysis of selenium methionine supplementation (0, 0.5, 1, and 2%) on sperm mortality (%) during storage at 25 °C.

Time Point	Linear (R^2^, *p*-Value)	Quadratic (R^2^, *p*-Value)	Cubic (R^2^, *p*-Value)
0 h	0.065 (0.423)	0.725 (0.003)	0.988 (<0.0001)
4 h	0.140 (0.230)	0.771 (0.001)	0.984 (<0.0001)
8 h	0.123 (0.264)	0.693 (0.005)	0.940 (<0.0001)
12 h	0.215 (0.129)	0.720 (0.003)	0.985 (<0.0001)
24 h	0.187 (0.161)	0.774 (0.001)	0.990 (<0.0001)

**Table 12:** Polynomial contrast analysis of selenium methionine supplementation (0, 0.5, 1, and 2%) on sperm viability (%) during storage at 25 °C.

Time Point	Linear (R^2^, *p*-Value)	Quadratic (R^2^, *p*-Value)	Cubic (R^2^, *p*-Value)
0 h	0.023 (0.636)	0.787 (0.001)	0.983 (<0.0001)
4 h	0.066 (0.420)	0.819 (<0.0001)	0.990 (<0.0001)
8 h	0.078 (0.379)	0.797 (0.001)	0.990 (<0.0001)
12 h	0.066 (0.421)	0.800 (0.001)	0.988 (<0.0001)
24 h	0.066 (0.421)	0.800 (0.001)	0.988 (<0.0001)

**Table 13:** Polynomial contrast analysis of selenium methionine supplementation (0, 0.5, 1, and 2%) on straight-line velocity (VSL, µm/s) during storage at 25 °C.

Time Point	Linear (R^2^, *p*-Value)	Quadratic (R^2^, *p*-Value)	Cubic (R^2^, *p*-Value)
0 h	0.001 (0.939)	0.639 (0.010)	0.966 (<0.0001)
4 h	0.002 (0.878)	0.663 (0.007)	0.947 (<0.0001)
8 h	0.025 (0.623)	0.711 (0.004)	0.927 (<0.0001)
12 h	0.009 (0.768)	0.648 (0.009)	0.930 (<0.0001)
24 h	0.030 (0.591)	0.597 (0.017)	0.928 (<0.0001)

**Table 14:** Polynomial contrast analysis of selenium methionine supplementation (0, 0.5, 1, and 2%) on curvilinear velocity (VCL, µm/s) during storage at 25 °C.

Time Point	Linear (R^2^, *p*-Value)	Quadratic (R^2^, *p*-Value)	Cubic (R^2^, *p*-Value)
0 h	0.004 (0.841)	0.649 (0.009)	0.954 (<0.0001)
4 h	0.006 (0.810)	0.708 (0.004)	0.960 (<0.0001)
8 h	0.005 (0.820)	0.660 (0.008)	0.953 (<0.0001)
12 h	0.005 (0.820)	0.660 (0.008)	0.953 (<0.0001)
24 h	0.000 (0.948)	0.658 (0.008)	0.947 (<0.0001)

**Table 15:** Polynomial contrast analysis of selenium methionine supplementation (0, 0.5, 1, and 2%) on average path velocity (VAP, µm/s) during storage at 25 °C.

Time Point	Linear (R^2^, *p*-Value)	Quadratic (R^2^, *p*-Value)	Cubic (R^2^, *p*-Value)
0 h	0.009 (0.765)	0.653 (0.009)	0.952 (<0.0001)
4 h	0.006 (0.815)	0.682 (0.006)	0.953 (<0.0001)
8 h	0.004 (0.841)	0.649 (0.009)	0.954 (<0.0001)
12 h	0.005 (0.820)	0.660 (0.008)	0.953 (<0.0001)
24 h	0.001 (0.939)	0.639 (0.010)	0.966 (<0.0001)

**Table 16:** Polynomial contrast analysis of selenium methionine supplementation (0, 0.5, 1, and 2%) on amplitude of lateral head displacement (ALH, µm) during storage at 25 °C.

Time Point	Linear (R^2^, *p*-Value)	Quadratic (R^2^, *p*-Value)	Cubic (R^2^, *p*-Value)
0 h	0.051 (0.482)	0.563 (0.024)	0.766 (0.007)
4 h	0.064 (0.429)	0.465 (0.060)	0.485 (0.132)
8 h	0.051 (0.482)	0.563 (0.024)	0.766 (0.007)
12 h	0.051 (0.482)	0.563 (0.024)	0.766 (0.007)
24 h	0.024 (0.628)	0.539 (0.031)	0.755 (0.008)

**Table 17:** Polynomial contrast analysis of selenium methionine supplementation (0, 0.5, 1, and 2%) on linearity index (LIN) during storage at 25 °C.

Time Point	Linear (R^2^, *p*-Value)	Quadratic (R^2^, *p*-Value)	Cubic (R^2^, *p*-Value)
0 h	0.301 (0.065)	0.512 (0.040)	0.563 (0.072)
4 h	0.579 (0.004)	0.919 (<0.0001)	0.998 (<0.0001)
8 h	0.002 (0.884)	0.643 (0.010)	0.952 (<0.0001)
12 h	0.512 (0.009)	0.569 (0.023)	0.595 (0.054)
24 h	0.028 (0.604)	0.746 (0.002)	0.959 (<0.0001)

**Table 18:** Polynomial contrast analysis of selenium methionine supplementation (0, 0.5, 1, and 2%) on straightness index (STR) during storage at 25 °C.

Time Point	Linear (R^2^, *p*-Value)	Quadratic (R^2^, *p*-Value)	Cubic (R^2^, *p*-Value)
0 h	0.002 (0.899)	0.657 (0.008)	0.956 (<0.0001)
4 h	0.001 (0.905)	0.628 (0.012)	0.948 (<0.0001)
8 h	0.021 (0.651)	0.524 (0.035)	0.930 (<0.0001)
12 h	0.004 (0.836)	0.632 (0.011)	0.919 (<0.0001)
24 h	0.001 (0.917)	0.641 (0.010)	0.905 (<0.0001)

**Table 19:** Effect of selenium methionine supplementation on acrosome integrity (%) during storage at 25 °C.

Treatment Group	0 h	4 h	8 h	12 h	24 h
Control	59.24 ± 0.37 ^d^	56.00 ± 0.57 ^d^	52.90 ± 0.85 ^d^	49.00 ± 0.57 ^d^	48.00 ± 0.57 ^d^
Selenium methionine 0.5%	65.16 ± 0.62 ^a^	62.47 ± 0.71 ^a^	59.69 ± 0.54 ^a^	55.43 ± 0.70 ^a^	50.00 ± 0.88 ^a^
Selenium methionine 1%	63.80 ± 0.81 ^b^	60.90 ± 0.58 ^b^	57.50 ± 0.44 ^b^	53.20 ± 0.63 ^b^	49.10 ± 0.40 ^b^
Selenium methionine 2%	62.50 ± 0.72 ^c^	59.30 ± 0.66 ^c^	55.80 ± 0.76 ^c^	51.70 ± 0.52 ^c^	48.50 ± 0.60 ^c^

Note: Different superscript letters (a–d) indicate significant differences among treatment groups within the same time point (*p* < 0.05). Values are mean ± SEM.

**Table 20:** Effect of selenium methionine supplementation on plasma membrane integrity (%) during storage at 25 °C.

Treatment Group	0 h	4 h	8 h	12 h	24 h	% Decline Rate at 24 h
Control	53.80 ± 0.39 ^d^	51.20 ± 0.59 ^d^	48.50 ± 0.87 ^d^	45.80 ± 0.58 ^d^	43.00 ± 0.59 ^d^	20.1%
Selenium methionine 0.5%	60.90 ± 0.64 ^a^	58.30 ± 0.73 ^a^	55.60 ± 0.55 ^a^	52.90 ± 0.71 ^a^	50.20 ± 0.89 ^a^	17.6%
Selenium methionine 1%	59.50 ± 0.83 ^b^	56.80 ± 0.59 ^b^	54.00 ± 0.45 ^b^	51.20 ± 0.64 ^b^	48.40 ± 0.42 ^b^	18.7%
Selenium methionine 2%	58.10 ± 0.73 ^c^	55.30 ± 0.67 ^c^	52.50 ± 0.77 ^c^	49.60 ± 0.53 ^c^	46.70 ± 0.61 ^c^	19.6%

Note: Different superscript letters (a–d) indicate significant differences among treatment groups within the same time point (*p* < 0.05). Values are mean ± SEM.

**Table 21:** Pearson correlation coefficients between functional, kinematic, and structural sperm parameters at 25 °C (*n* = 20).

Parameter	VSL	VCL	VAP	ALH	LIN	STR	Acrosome Integrity	Plasma Membrane Integrity
Total sperm motility	r = 0.952 (*p* < 0.001)	r = 0.990 (*p* < 0.001)	r = 0.942 (*p* < 0.001)	r = 0.982 (*p* < 0.001)	r = 0.921 (*p* < 0.001)	r = 0.929 (*p* < 0.001)	r = 0.847 (*p* < 0.001)	r = 0.948 (*p* < 0.001)
Sperm viability	r = 0.975 (*p* < 0.001)	r = 0.990 (*p* < 0.001)	r = 0.958 (*p* < 0.001)	r = 0.988 (*p* < 0.001)	r = 0.950 (*p* < 0.001)	r = 0.962 (*p* < 0.001)	r = 0.857 (*p* < 0.001)	r = 0.946 (*p* < 0.001)
Mortality	r = −0.952 (*p* < 0.001)	r = −0.990 (*p* < 0.001)	r = −0.942 (*p* < 0.001)	r = −0.982 (*p* < 0.001)	r = −0.921 (*p* < 0.001)	r = −0.929 (*p* < 0.001)	r = −0.847 (*p* < 0.001)	r = −0.948 (*p* < 0.001)

## Data Availability

The original contributions presented in this study are included in the article. Further inquiries can be directed to the corresponding author(s).
